# Efficacy of Intravitreal injection of 2-Methoxyestradiol in regression of neovascularization of a retinopathy of prematurity rat model

**DOI:** 10.1186/s12886-017-0433-3

**Published:** 2017-04-04

**Authors:** Azza Mohamed Ahmed Said, Rania Gamal Eldin Zaki, Rania A. Salah Eldin, Maha Nasr, Samar Saad Azab, Yaser Abdelmageuid Elzankalony

**Affiliations:** 1 Ophthalmology Department, Faculty of Medicine, Ain Shams University, Cairo, Egypt; 2 Anatomy and Embryology Department, Faculty of Medicine, Ain Shams University, Cairo, Egypt; 3 Department of Pharmaceutics and Industrial Pharmacy, Faculty of Pharmacy, Ain Shams University, Cairo, Egypt; 4 Department of Pharmacology and Toxicology, Faculty of Pharmacy, Ain Shams University, Cairo, Egypt; 5 10th Fawzy Elmoteay Street, Heliopolis, Cairo 11361 Egypt

**Keywords:** 2-methoxyestradiol, Neovascularization, VEGF, GFAP, Retinopathy of prematurity

## Abstract

**Background:**

Retinopathy of prematurity (ROP) is one of the targets for early detection and treatment to prevent childhood blindness in world health organization programs. The purpose of study was to evaluate the efficacy of intravitreal injection of 2-Methoxyestradiol (2-ME) nanoemulsion in regressing neovascularization of a ROP rat model.

**Methods:**

A prospective comparative case - control animal study conducted on 56 eyes of 28 healthy new born Sprague Dawley male albino rat. ROP was induced in 21 rats then two concentrations of 2-ME nanoparticles were injected in right eyes of 14 rats (low dose; study group I, high dose; study group II). A blank nanoemulsion was injected in the right eyes of seven rats (control positive group I). No injections performed in contralateral left eyes (control positive group II). Seven rats (14 eyes) were kept in room air (control negative group). On postnatal day 17, eyeballs were enucleated. Histological structure of the retina was examined using Hematoxylin and eosin staining. Vascular endothelial growth factor (VEGF) and glial fibrillary acidic protein (GFAP) expressions were detected by immunohistochemical studies.

**Results:**

Intravitreal injection of 2-ME (in the two concentrations) caused marked regression of the new vascular tufts on the vitreal side with normal organization and thickness of the retina especially in study group II, which also show negative VEGF immunoreaction. Positive GFAP expression was detected in the control positive groups and study group (I).

**Conclusion:**

Intravitreal injection of 2-Methoxyestradiol nanoemulsion is a promising effective method in reduction of neovascularization of a ROP rat model.

**Electronic supplementary material:**

The online version of this article (doi:10.1186/s12886-017-0433-3) contains supplementary material, which is available to authorized users.

## Background

Retinopathy of prematurity (ROP) is a disease affects the retina of premature infants. Retinal neovascularization (NV), occurs due to local ischemia. In the more serious types of the disease, the abnormal vascular changes advance to retinal detachment. Once retinal detachment happens, the prognosis for visual recovery is poor [1, 2]. ROP is a main cause of preventable childhood retinal dysfunction. The World Health Organization’s Vision 2020 program targets ROP as an avoidable disease requiring early detection and treatment to counteract visual deficiency [3].

The most popular model to study abnormal angiogenesis in the retina is the oxygen-induced retinopathy (OIR) model in mice produced by Smith et al. [4]. They exposed one - week old mouse pups to hyperoxia. Hyperoxia obliterates capillaries in the retina. Upon return to room air, the retina becomes hypoxic and triggers a repair response, which then results in the formation of neovascular tufts towards the vitreous. This is an important sign of ischemic retinopathies in human pathologies. The tuft formation is known as ‘pathological angiogenesis’ and makes the OIR model a key tool in study of vascular pathology in ischemic retinopathies [4]. The model by Smith et al. [5] becomes the protocol of choice because it is reproducible and easily quantifiable [6, 7].

The OIR model simulates events occur during ROP, including the pathological alterations that influence premature infants. At postnatal day 18, significant changes occurred in both the retinal vasculature and neural function in that model [2, 8].

Owing to excess production of vascular endothelial growth factor (VEGF); retinal vascular permeability is higher in the rat OIR model than in normal rats. In addition, degeneration of astrocytes was included in the failure of the blood retinal barrier [9].

2-Methoxyestradiol (2-ME) is a biologically active metabolite of 17 B - estradiol, inhibits key processes associated with cell replication in vitro. It may have powerful growth-inhibitory effects on proliferating cells, including smooth muscle cells and endothelial cells and may be antiangiogenic in vivo [10].

This study aimed to evaluate the efficacy of intravitreal injection of 2-ME nanoemulsion with various concentrations in causing regression of NV in OIR rat’s model.

## Methods

### Study design

A prospective comparative case - control animal study was conducted at Ophthalmology, Anatomy and Embryology Departments and the Medical Research Center, Faculty of Medicine, Ain Shams University in the period from April 2014 to November 2014.

All experimental procedures conformed to the guidelines provided by the CPCSEA for studies and the ARVO resolution on the use of animals in research and to institutional guidelines. The study performed according to the recommendations of Faculty of Medicine, Ain Shams University Research Ethical Committee, (FMASU REC).

Twenty eight healthy new born Sprague Dawley male albino rats were locally bred at the animal house of the Medical Research Center. Rats were housed in stainless steel cages, two rats per cage, the size of each cage was 30 × 35 cm, and were left for a week before any intervention to acclimatize to experimental conditions. The rats were allowed daily diet and free water access (ad libitum) with suitable environmental conditions and good ventilation. ROP was induced in 21 rats then two concentrations of 2-ME (Sigma-Aldrich, Germany) were injected in right eyes of 14 rats (low dose; study group I, high dose; study group II). A blank nanoemulsion was injected in the right eyes of seven rats (control positive group I). No injections were performed in contralateral left eyes (control positive group II). The remaining seven rats (14 eyes) were kept in room air without exposure to high levels of oxygen or medications (control negative group).

### Experimental protocol

#### Induction of retinopathy of prematurity

ROP model developed by Smith et al. [5] was used in this study. Newborn Sprague Dawley rats were exposed to a mean of 75% oxygen from postnatal day 7 to postnatal day 12, with their nursing mothers in a special incubator [11]. Delivery of this high percentage of oxygen to the incubator was performed using a portable oxygen concentrator instrument known as Air Sep New Life Elite and intensity combines high pressure with high flow (Air Sep Corporation, Buffalo, New York, USA). At postnatal day 12, the animals were returned to room air (21% oxygen) [12].

#### Preparation of the blank nanoemulsion

500 μl Labrafac Lipophile oil (Gattefosse’ company, France) added 50 ml phosphate buffered saline pH 7.4 containing 0.1% tween 80 (El Nasr pharmaceutical company, Egypt).

#### Preparation of 2-Methoxyestradiol nanoemulsion

2-ME was prepared in nanoemulsion form using high shear homogenization method. The drug was dissolved in 500 μl Labrafac Lipophile oil (Gattefosse’ company, France) with the aid of 1 ml ethanol, followed by addition to 50 ml phosphate buffered saline pH 7.4 containing 0.1% tween 80 (El Nasr pharmaceutical company, Egypt). Homogenization of the nanoemulsion contents was performed at 24,000 rpm (Heidolph DIAX 900, Germany), followed by magnetic stirring for 2 h to ensure complete evaporation of ethanol. The nanoemulsion was characterized for particle size and polydispersity index after appropriate dilution using (Zetasizer, Malvern Instruments, UK) and for morphology using transmission electron microscope (JEM-100 S, Joel, Japan) after negative staining with uranyl acetate (Allied Signal company, Germany).

**Fig. 1:**
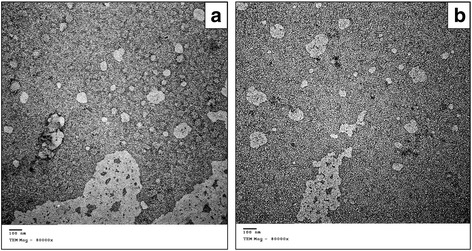
Transmission electron microscope micrographs of **a** 10 μg/ml and **b** 100 μg/ml of 2- Methoxyestradiol nanoemulsions. Both displayed a particle size of 161 nm and a polydispersity index of 0.23, indicating monodisperse homogenous formulae (original magnification X80000)

Regarding 2-ME nanoemulsion formulations, the low and high dose nanoemulsions both displayed a particle size of 161 nm and a polydispersity index of 0.23, indicating monodisperse homogenous formulae. Transmission electron microscope micrographs of the low and high dose 2- ME nanoemulsions are demonstrated in (Fig. 1a, b).

#### Intravitreal 2-Methoxyestradiol injection for therapy

Intravitreal injection was performed on postnatal day 12 under complete sterile conditions [11]. This was started by intraperitoneal injection of a combination of ketamine hydrochloride [(KETAM, Egyptian International pharmaceutical industries company E.I.P.I.CO) (40 mg/kg), and xylazine hydrochloride (5 mg/ml)] in order to deeply anaesthetized the newborn rats. The eyes were topically anaesthetized by a drop of Benoxinate Hydrochloride ophthalmic solution 0.4% (Egyptian Int. Pharmaceutical Industries Co. [E.P.I.CO]).

Under a stereoscopic operating microscope, the upper nasal part of the sclera of the right eye was pierced 1.5 mm behind the limbus using a 30-gauge insulin needle fitted to a syringe containing the calculated volume of steroid in 0.02 ml, after the pupil was dilated with Tropicamide 1% eye drops. The needle tip was observed during the procedure to avoid retinal injury. Injection of the steroids or blank nanoemulsion was performed into the vitreous over 3 min to avoid reflux. The right eyes of OIR rat’s model (21 rats) were divided into the following groups:

- **Control positive group I**: seven eyes were injected with the blank nanoemulsion.
- **Study group I (low dose 2-Methoxyestradiol treated group)**: seven eyes received 0.02 ml of low dose 2-ME nanoemulsion (10 μg/ml).
- **Study group ІІ** (**High dose 2-Methoxyestradiol treated group)**: seven eyes received 0.02 ml of high dose 2-ME nanoemulsion (100 μg/ml).

### Histopathology

On postnatal day 17, all animals were sacrificed under deep general anesthesia (intraperitoneal injection of the same drugs used for anesthesia at the time of intravitreal injections using double the dose) then decapitation was performed. After enucleation, the eyeballs were dissected and cut coronally into anterior and posterior segments. The anterior and the posterior segments that containing the retina were further divided sagittally into two parts. The peripheral retina of each part was obtained to get away from the central retina. Specimens were immediately fixed in 10% neutral formalin, and then were processed for paraffin blocks. The specimens were cut into sections of 5–7 um in thickness. Sections were stained with hematoxylin and eosin according to Drury and Wallington [13] to examine the histological structure of the retina in the different groups. The anterior segments were examined for drug toxicity.

### Immunohistochemistry

Every fifth paraffin section was mounted on polylysine-coated slides or on charged slides to be immunohistochemically stained. To detect vascular integrity and endothelial function, immunohistochemical staining for vascular endothelial growth factor (VEGF) was performed according to Youssef and Said [14]. To detect the glial cells reactivity, immunohistochemical staining for glial fibrillary acidic protein (GFAP) was performed according to Chen and Weber [15].

### Histological morphometric analysis

The percentage of the area of positive staining of each of GFAP and VEGF was calculated in the different groups using a computer image analyzer Leica Q 500 MC program. The computer was connected to an Olympus microscope (model BX51, Olympus Japan) equipped with digital camera for histological grading at (magnification X400) [14]. The measurements were done in four non overlapping fields in each of five different sections taken from all animals of each group.

Endothelial cell nuclei on the vitreal side of internal limiting membrane (ILM) were count in masked fashion by one of the authors. Counts were recorded from six images of each retinal specimen (magnification X400) from every group and the average number was taken in each group.

### Statistical analysis

All data were collected and analyzed statistically using social package for statistical science (SPSS) for windows version 13.0 (SPSS Inc., Chicago, USA). Quantitative data were expressed as mean and standard deviation. Student-t test was used for comparison of quantitative variables between two groups. One - way analysis of variance (ANOVA) was employed to compare means between more than two groups. The significance of the data was determined by the probability (*P*-value). *P* > 0.05 was considered insignificant, *P* ≤ 0.05 was considered significant, and *P* ≤ 0.01 was considered highly significant (Additional file 1).

## Results

Upon clinical examination of all rats, endophthalmitis was noticed 48 h following intravitreal injection in one eye (14.3%) of the study group (I). This eye was not subjected to histopathological examination. Cataract was developed in one eye (14.3%) of the study group (I) and two eyes (28.6%) of the study group (II). No histological abnormalities were noted in the ciliary body or the cornea in any of the studied groups.

**Fig. 2:**
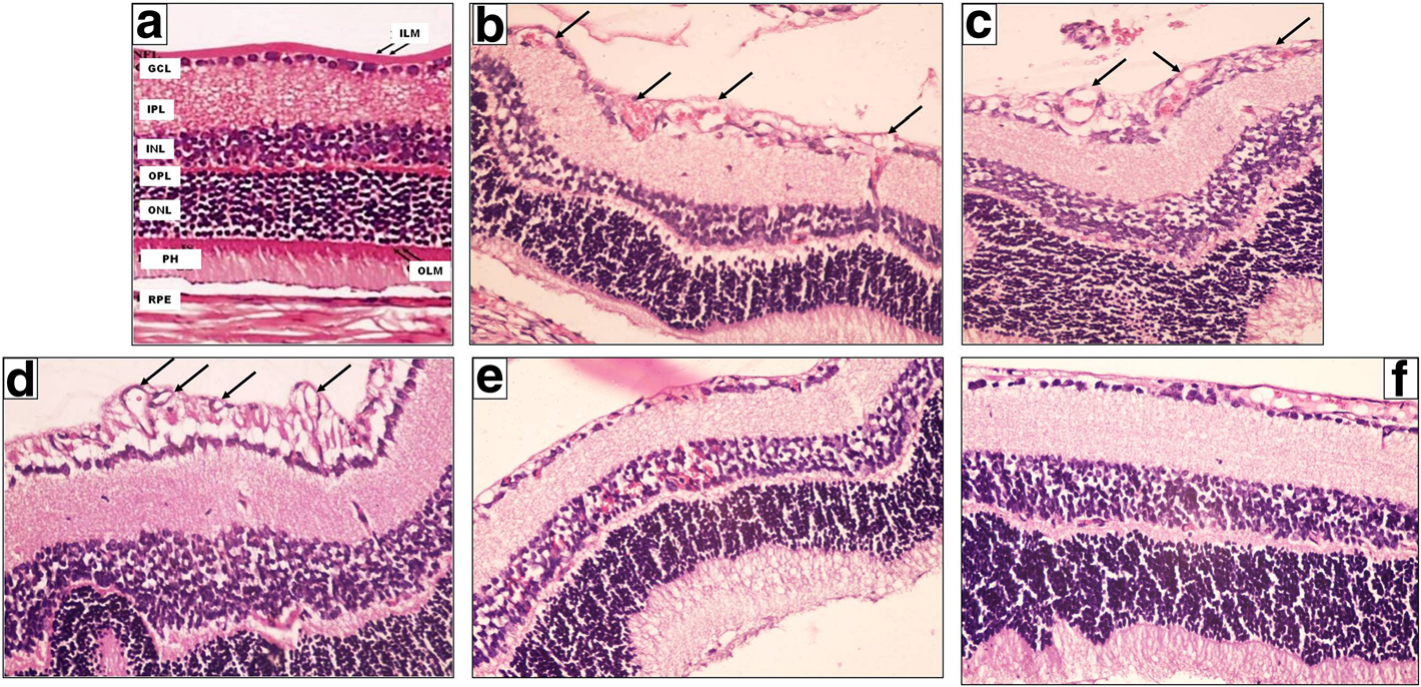
A photomicrograph of a section of a rat’s retina. **a** Normal arrangement of the different retinal layers in the control negative group (room air). **b**, **c** Control positive group (I) and (II) respectively show disorganization in ONL and INL. It also shows the presence of neovascularization both in INL and on the vitreal side of ILM (*black arrows*). **d** Another photo of the control positive group (II) shows well apparent neovascularization both in INL and on the vitreal side of ILM (*black arrows*). **e** Eye received intravitreal injection of 0.02 ml of 2-Methoxyestradiol (10 μg/ml) shows mild regression of superficial and deep neovessels **f** Eye received intravitreal injection of 0.02 ml of 2-Methoxyestradiol (100 μg/ml) shows regression of superficial and deep neovessels and preservation of the normal structure of the retina (Hematoxylin-eosin, original magnification (original magnification X400)

The histological picture of rat’s retina of the control negative group showed the normal arrangement of the ten layers of the retina (Fig. 2a).

In the control positive groups (I) and (II), the histological changes were similar; irregular appearance of the photoreceptor (PH) layer with loss of fibrillary parallel striations. In some sections, it showed many vacuoles and focal loss. There was decrease in cell density within the outer nuclear layer (ONL) resulting in spacing between the cells. The outer plexiform layer (OPL) lost its normal appearance and showed marked distortion with widening of the spaces between the fibers giving a spongiform appearance. Retinal vessels appeared within this layer. The inner nuclear layer (INL) showed decrease in cell density and spacing between the cells. Some vessels appeared in this layer.

The inner plexiform layer (IPL) showed widening of the spaces between the fibers giving a spongiform appearance. Marked disorganization of the ganglion cell layer (GCL) was noticed compared to the control negative group with decrease in cellular density. Blood vessels were observed in this layer. A decrease in the nerve fiber layer (NFL) thickness was observed. In some sections, ILM was preserved and appeared as an intact continuous membrane which represents the innermost layer of the retina. However, in other sections, it was not apparent (Fig. 2b, c, d).

### Quantification of proliferative retinopathy

The control negative group showed the normal retinal pattern, at postnatal day 17 which is consisting of branching superficial vessels connecting with a deep vascular layer that extended from the optic nerve to the ora serrata. Vascular cells, stained with hematoxylin - eosin, did not extend beyond the ILM into the vitreous. The eyes of rats exposed to hyperoxia from postnatal day 7 to postnatal day 12 and returned to room air until postnatal day 17 (control positive group II) had neovascular tufts, extending beyond the ILM into the vitreous with similar results in (control positive group I) (Fig. 2d).

NV as a result of hyperoxia was quantified in serial paraffin cross sections by counting the number of vascular cell nuclei on the vitreal side of the ILM. In normal postnatal day 17 rats, no nuclei was detected on the vitreal side of ILM per 6 μm retinal cross-section compared to 17.04 nuclei ±2.31 Standard deviation (SD) (range: 13.0–21.0) per 6 μm cross-section in the hyperoxia- exposed eyes. Although the profiles of the deeper vessels were not numerous per 6 μm cross-section in the hyperoxia-exposed retinas, the average profile appeared greater in cross-sectional area and stained more heavily with hematoxylin – eosin than did the vascular profiles in the normal retina. The mean endothelial cell nuclei number on the vitreal side of ILM in the nanoemulsion group (control positive group (I) was 15.85 nuclei ±2.41 SD (range: 13.0–20.0) with no statistically significant difference between it and the OIR control positive group (II) (*P*-value was 0.27).

The mean endothelial cell nuclei number on the vitreal side of ILM in the study group (I) was 4.17 nuclei ±1.47 SD (range: 3.0–6.0) and it was 2.28 nuclei ±1.6 SD (range: 0.0–4.0) in the study group (II). There was a highly statistically significant increase in the mean number of nuclei in the control positive groups and the other study groups compared to the control negative group (*P*-value <0.01). The mean number started to decrease in the study groups when compared to the control positive groups (I) and (II) and this reduction was highly statistically significant (*P*-value <0.01).

Comparison was done within the study groups revealed no statistically significant difference between study groups (I) and (II) (*P*-value =0.08). Compared to the control positive groups (I) and (II), endothelial cell nuclei were reduced in the study groups with highest percentage of suppression in the study group (II). The percentage of suppression of endothelial cell nuclei on the vitreal side after therapy was 75.6% in study group (I) and 86.6% in study group (II).

In response to intravitreal injection of 2-ME; there was marked regression of the new vascular tufts on the vitreal side with apparent normal organization and thickness of the ONL and INL compared to the control positive group. This was more evident in study group (II) (Fig. 2e, f).

### Immunohistochemical results

#### VEGF immunostaining

**Fig. 3:**
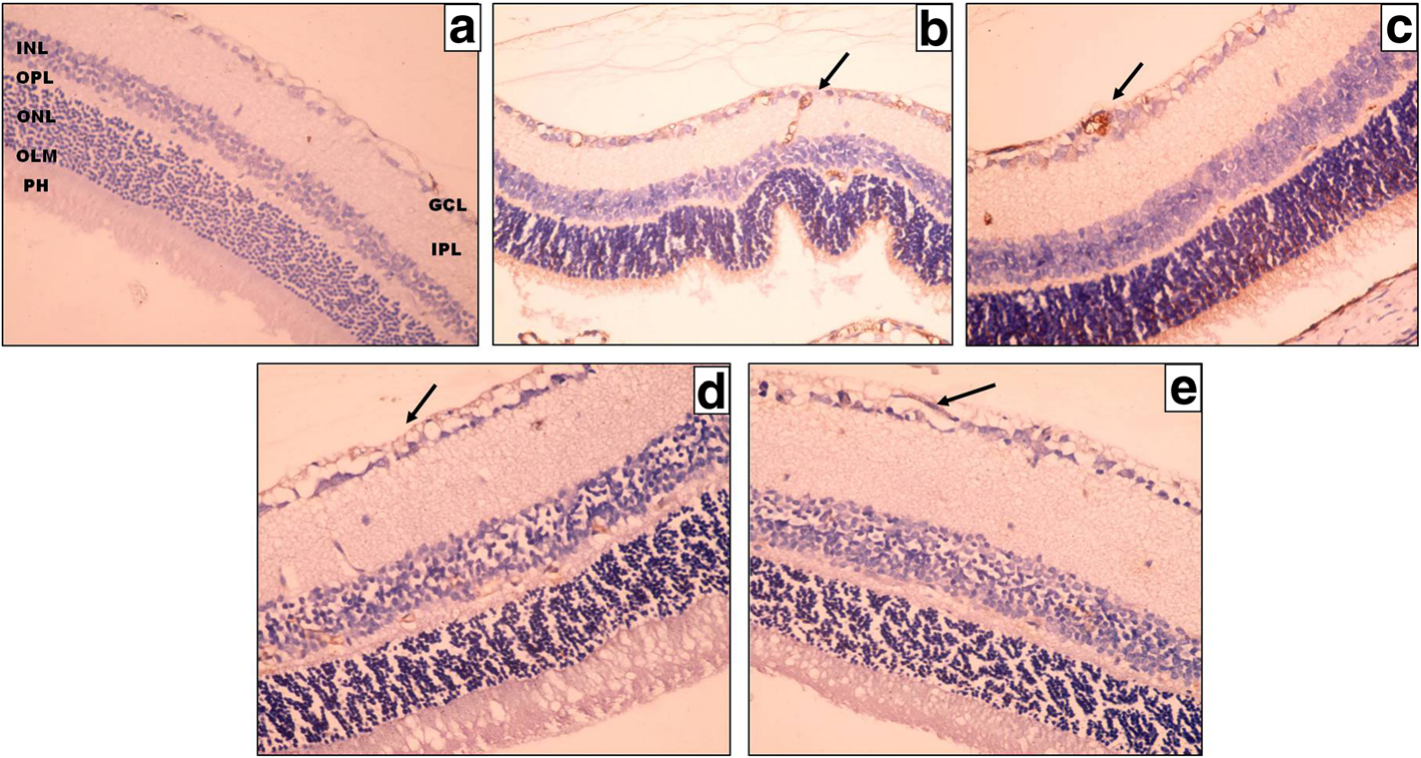
A photomicrograph of a section of a rat’s retina. **a** Control negative group shows negative VEGF immunohistochemical staining in all the retinal layers. **b**, **c** Control positive group (I) and (II) respectively show faint VEGF immunohistochemical staining in few cells; in GCL, ONL and INL (*black arrow*). **d** Eye received intravitreal injection of 0.02 ml of 2-Methoxyestradiol (10 μg/ml). **e** Eye received intravitreal injection of 0.02 ml of 2-Methoxyestradiol (100 μg/ml) shows weak positive VEGF immunohistochemical staining in all retinal layers (*black arrow*) (original magnification X400)

The control negative group revealed negative VEGF immunohistochemical staining in all the retinal layers (Fig. 3a). The control positive groups and the study group (I) showed faint VEGF immunohistochemical staining in few cells in GCL, INL, and ONL (Fig. 3b, c, d). In study group (II) the retina showed negative VEGF immunoreaction in all the retinal layers (Fig.3 e). The mean percentage of area of immunohistochemical staining of VEGF in the control positive (I), control positive (II), study group (I) and study group (II) was 0.015, 0.02, 0.03 and 0.07 respectively.

#### GFAP immunostaining

**Fig. 4:**
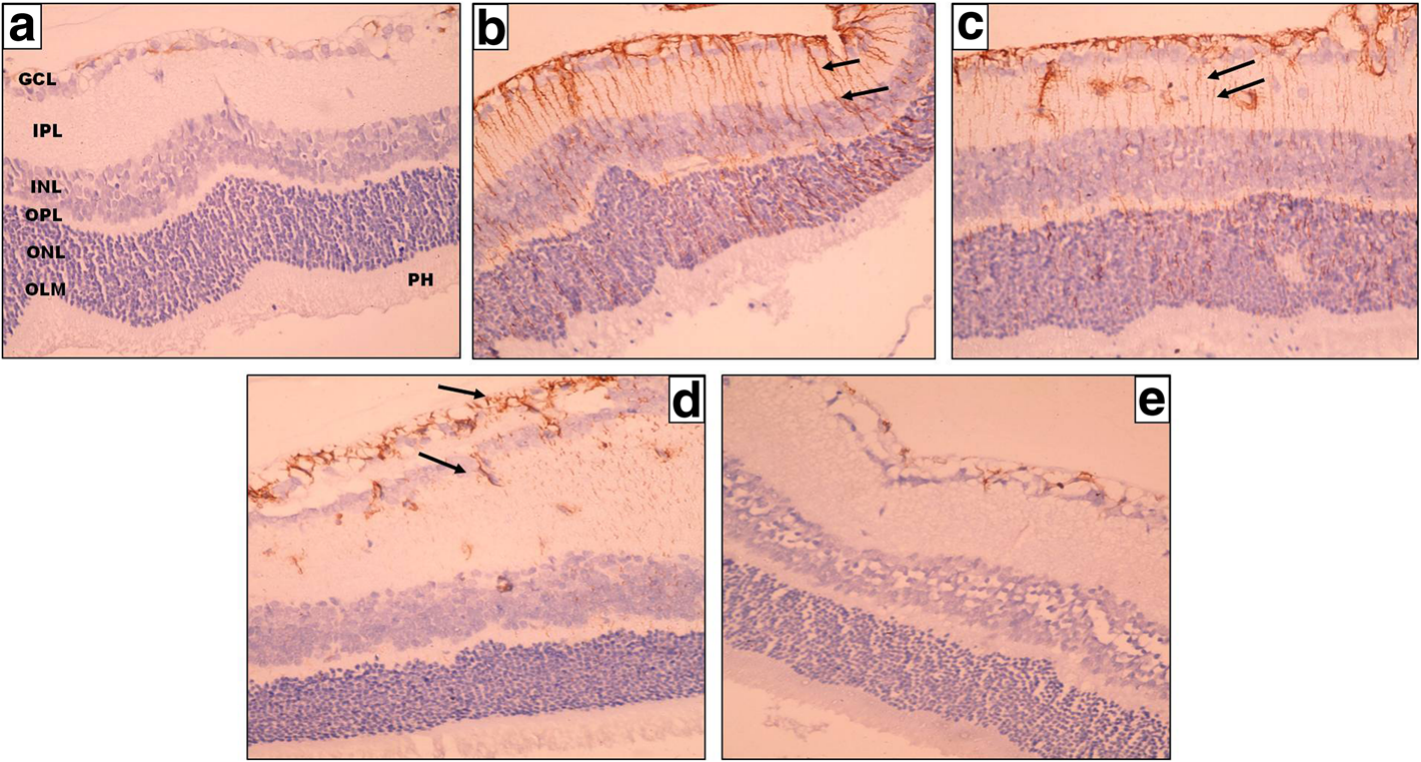
A photomicrograph of a section of a rat’s retina. **a** Control negative group shows localized positive GFAP immunohistochemical staining confined to GCL, NFL, and ILM. **b**, **c** Control positive group (I) and (II) respectively show positive GFAP staining of Muller cells radial processes extended throughout the whole retinal thickness; from the ILM to the OLM (*black arrows*). **d** Eye received intravitreal injection of 0.02 ml of 2-Methoxyestradiol (10 μg/ ml) and (**e**) Eye received intravitreal injection of 0.02 ml of 2-Methoxyestradiol (100 μg/ml) showing limited GFAP expression confined only to GCL, NFL, and ILM (*black arrows on*) **d** (original magnification X400)

In the control negative group, The GFAP labeling was mostly localized in GCL, NFL and ILM. Other layers showed negative GFAP immunohistochemical staining (Fig.4a). The intensity of the GFAP expression was increased in the control positive groups and study group (I) compared to the control negative group. The marked increase in GFAP immunohistochemical staining appeared as stained Muller cells radial processes extended throughout the whole retinal thickness and even between the photoreceptors (Fig. 4b, c).

In the study group (I); GFAP immunohistochemical staining appeared to accompany the distribution of Muller cells radial processes in the inner retinal layers; IPL, GCL, NFL, and ILM (Fig. 4 d). Some stained processes were seen traversing between the cells of the INL. Mild GFAP expression was detected in OPL.

Study group (II) showed similar results as the control negative group. They showed limited GFAP expression confined only to GCL, NFL, and ILM (Fig. 4e). The mean percentage of area of immunohistochemical staining of GFAP in the control negative group, control positive group (I), control positive group (II), study group (I) and study group (II) group was 2.24, 15.24, 15.82, 11.39 and 4.92 respectively.

There was a highly statistically significant difference as regards the mean percentage of area of GFAP immunostaining between all groups (*P*-values <0.01). Comparison between the control positive groups and each of the study groups revealed a highly statistically significant reduction in percentage of area of expression in the study groups (I and II) compared to control positive groups (*P*-values <0.01). When comparison was done between the study groups; a highly statistically significant reduction more evident in study group (II) compared to study group (I) (*P*-values <0.01).

## Discussion

It is well known that newly formed capillaries in the retina of premature infants and some species of newborn animals are sensitive to oxygen, a feature that predisposes them to ROP [16]. 2-ME is an antiangiogenic agent, has low estrogenic activity and no cytotoxic effects. So it could be a valuable therapeutic molecule for prevention and treatment of neovascular ocular disorders like ROP [10]. There is no previous research about the effect of this molecule on ROP before so here the effect of 2-ME with various concentrations on vascular activity and angiogenesis was evaluated and also its effect in causing regression of NV in OIR rat’s model.

A major challenge of developing 2-ME as a useful drug is overcoming its poor oral bioavailability and short half-life. 2-ME may be less efficacious and potent in vivo compared with its actions in vitro [10]. Nanotechnology and drug modeling is a key in resolving the pharmacological aspects that reduce the therapeutic potential of 2-ME. In such manner, modification of the molecule to target particular tissues will also be helpful to enhance its therapeutic potential and reduce adverse effects [17].

So in this study 2- ME was prepared in nanoemulsion form using high shear homogenization method to overcome these undesirable pharmacokinetic properties. In response to intravitreal injection of 2-ME; there was marked regression of the new vascular tufts on the vitreal side with apparent normal organization and thickness of the ONL and INL compared to the control positive groups. This was more evident in study group (II). These results show that intravitreal 2-ME 100 μg/ml has better antiangiogenic effect on the neovascular vessels decreasing endothelial proliferation.

There is no previous study about the effect of 2-ME on endothelial proliferation in OIR rat’s model but there are studies about other materials [11, 18–21].

Several studies [22–27] addressed the antiangiogenic effects of 2-ME in several diseases. Oral administration of 2-ME inhibits angiogenesis in vivo and suppresses systemic tumor growth by limiting blood supply. 2-ME at high concentrations was used in pulmonary hypertension, rheumatoid arthritis, systemic tumors and endometriosis [10].

It interacts with endothelial tubulin dynamics and hypoxia inducible factor − 1 alpha to affect target proteins and genes involved in processes regulate angiogenesis [10].

Robinson et al. [28] determined the safety and pharmacokinetics of sustained-release intravitreal 2-ME implants in normal rabbit and test their efficacy in a rat model of choroidal neovascularization (CNV). No ocular toxicities were reported by clinical examination, electroretinography and histopathology. Vitreous levels of 2-ME were within the therapeutic range for the inhibition of endothelial cell proliferation. A significant reduction in CNV in eyes treated with the 2-ME implant was detected.

Funakoshi et al. [29] evaluated the efficacy of systemic 2-ME in a laser-induced murine model of CNV. They found that the 2-ME decreased CNV in a dose - dependent manner. No toxicity or weight loss was observed during the treatment. Significant antiangiogenic effects of oral 2-ME on laser induced CNV were observed.

VEGF signaling pathway is a dominant pathway in conditions in which hypoxia occurs, however it is not the only pathway involved in the development of intravitreal NV [30, 31]. The results of the present study show that control negative group revealed negative VEGF immunohistochemical staining in all the retinal layers. The control positive groups and the study group (I) showed faint VEGF immunohistochemical staining in few cells in GCL, INL, and ONL. The study group (II) showed negative expression of VEGF.

The faint VEGF immunohistochemical staining could be also explained by Chan-Ling and Stone findings [32]. It is possible that a combination of loss of astrocytes and a shift in angiogenic growth factor expression to the inner retina are critical factors for the aberrant growth of blood vessels into the vitreous [1].

Gliosis as demonstrated by upregulation of the intermediate filament GFAP. It is upregulated in Muller cells indicating abnormal macroglial cells in ROP. Moreover, the upregulation of GFAP is restricted to the peripheral avascular retina, and also regions of the mid peripheral retina that are devoid of blood vessels. These findings suggest that Muller cells are altered in response to the reduction in retinal vascularization and are a response to localized retinal stress [1, 32].

The present study demonstrated that 2-ME in a dose of 100 μg/ml has an effective role in decreasing gliosis associated with NV. Pathological neovascularization, retinal degeneration and glial activation were the main mechanisms involved in ROP [33, 34].

The histopathological analysis shows that 2-ME both low and high doses have no toxic effect on the cornea, ciliary body. Three cases of cataract development following 2-ME injection were reported in the present study despite previous reports of weak protective association between estradiol and development of cataract [35]. Hales et al. [36] stated that sex-dependent and estrogen-related differences in susceptibility to cataract formation are consistent with a protective role for estrogen. Chen et al. [37] supported that treatment by 17 B-estradiol prevents induced cataracts in female rat lenses but not in males. Hormonal replacement therapy might reduce the risk of lens opacity [38]. It is still a matter of debate whether exogenous estrogen is protective against cataract development or not [36].

## Conclusion

2-Methoxyestradiol nanoemulsion is a promising effective agent in reduction of neovascularization of a ROP rat’s model. Additional and more detailed studies are needed to determine the appropriate dosage and time of intravitreal administration of 2-Methoxyestradiol for management of ROP in human.

## Additional file

Additional file 1: Data.xlsx includes raw data of ROP animal models that were injected inravitreally using two different concentrations of 2-Methoxyestradiol (2-ME) nanoparticles in order to produce regression of neovascularization (NVs) and the data of the control groups. The file includes one data sheet. The sheet includes data of each animal in terms of endothelial cell count on vitreal side, percentage of vascular endothelial growth factor (VEGF) expression and percentage of glial fibrillary acidic protein (GFAP) expression before and after treatment. (XLSX 12 kb)

## Abbreviations

2-ME: 2-Methoxyestradiol
ANOVA: Analysis of variance
ARVO: Association of Research in Vision and Ophthalmology
CNV: Choroidal neovascularization
CPCSEA: Committee for the Purpose of Control and Supervision of Experiments on Animals
GCL: Ganglion cell layer
GFAP: Glial fibrillary acidic protein
ILM: Internal limiting membrane
INL: Inner nuclear layer
IPL: Inner plexiform layer
NFL: Nerve fiber layer
NV: Neovascularization
OIR: Oxygen induced retinopathy
ONL: Outer nuclear layer
OPL: Outer plexiform layer
PH: Photoreceptor
*P*-value: Probability
ROP: Retinopathy of prematurity
SD: Standard deviation
SPSS: Social package for statistical science
VEGF: Vascular endothelial growth factor
